# Supplementary material for: Resolving the full spectrum of human genome variation using Linked-Reads
Source: Genome Res. 2019 Apr;29(4):635–45. doi: 10.1101/gr.234443.118 (PMC6442396; doi:10.1101/gr.234443.118)
Supplement: Supplemental Material [file supp_gr.234443.118_Supplemental_longranger-2.2.zip › longranger-2.2.tar.bz2!/lr2.2-public/lib/stan/cmdstan/stan/doc/api/html/functions_vars_d.html]

Stan: Class Members - Variables


|  |  |
| --- | --- |
|  | Stan  2.5.0  probability, sampling & optimization |


- Main Page
- Related Pages
- Namespaces
- Classes
- Files

- Class List
- Class Index
- Class Hierarchy
- Class Members

- All
- Functions
- Variables
- Typedefs
- Enumerator
- Related Functions

- \_
- a
- b
- c
- d
- e
- f
- g
- h
- i
- k
- l
- m
- n
- o
- p
- q
- r
- s
- t
- u
- v
- w
- x
- y
- z


All Classes Namespaces Files Functions Variables Typedefs Enumerations Enumerator Friends Macros Pages

### - d -

- d\_
  : stan::agrad::fvar< T >
- d\_x1
  : stan::agrad::OperandsAndPartials< T1, T2, T3, T4, T5, T6, T\_return\_type >
- d\_x2
  : stan::agrad::OperandsAndPartials< T1, T2, T3, T4, T5, T6, T\_return\_type >
- d\_x3
  : stan::agrad::OperandsAndPartials< T1, T2, T3, T4, T5, T6, T\_return\_type >
- d\_x4
  : stan::agrad::OperandsAndPartials< T1, T2, T3, T4, T5, T6, T\_return\_type >
- d\_x5
  : stan::agrad::OperandsAndPartials< T1, T2, T3, T4, T5, T6, T\_return\_type >
- d\_x6
  : stan::agrad::OperandsAndPartials< T1, T2, T3, T4, T5, T6, T\_return\_type >
- da\_
  : stan::agrad::precomp\_v\_vari
- data
  : stan::io::stan\_csv\_metadata
- data\_decl\_
  : stan::gm::program
- data\_var\_decls\_r
  : stan::gm::program\_grammar< Iterator >
- decl\_
  : stan::gm::var\_decl
- decl\_defs\_
  : stan::gm::function\_decl\_defs
- declare\_vars\_
  : stan::gm::generate\_local\_var\_init\_nan\_visgen
  , stan::gm::init\_local\_var\_visgen
- delta\_
  : stan::mcmc::stepsize\_adaptation
- depth\_
  : stan::mcmc::base\_nuts< M, P, H, I, BaseRNG >
- derived\_data\_decl\_
  : stan::gm::program
- derived\_data\_var\_decls\_r
  : stan::gm::program\_grammar< Iterator >
- derived\_decl\_
  : stan::gm::program
- derived\_var\_decls\_r
  : stan::gm::program\_grammar< Iterator >
- digits
  : std::numeric\_limits< stan::agrad::fvar< T > >
  , std::numeric\_limits< stan::agrad::var >
- digits10
  : std::numeric\_limits< stan::agrad::fvar< T > >
  , std::numeric\_limits< stan::agrad::var >
- dims\_
  : stan::gm::base\_var\_decl
  , stan::gm::variable\_dims
- dims\_r
  : stan::gm::statement\_grammar< Iterator >
  , stan::gm::term\_grammar< Iterator >
  , stan::gm::var\_decls\_grammar< Iterator >
- dimss\_
  : stan::gm::index\_op
- dist\_
  : stan::gm::sample
- distribution\_r
  : stan::gm::statement\_grammar< Iterator >
- double\_decl\_r
  : stan::gm::var\_decls\_grammar< Iterator >
- double\_literal\_r
  : stan::gm::term\_grammar< Iterator >
- dtrs\_
  : stan::agrad::stored\_gradient\_vari


---

[ Stan Home Page ]

*© 2011–2014,
Stan Development Team.*
